# Effects of six‐month creatine supplementation on patient‐ and clinician‐reported outcomes, and tissue creatine levels in patients with post‐COVID‐19 fatigue syndrome

**DOI:** 10.1002/fsn3.3597

**Published:** 2023-09-20

**Authors:** Jelena Slankamenac, Marijana Ranisavljev, Nikola Todorovic, Jelena Ostojic, Valdemar Stajer, Sergej M. Ostojic

**Affiliations:** ^1^ Applied Bioenergetics Lab, Faculty of Sport and PE University of Novi Sad Novi Sad Serbia; ^2^ Faculty of Medicine University of Novi Sad Novi Sad Serbia; ^3^ Department of Nutrition and Public Health University of Agder Kristiansand Norway; ^4^ Faculty of Health Sciences University of Pecs Pecs Hungary

**Keywords:** creatine, post‐COVID‐19 fatigue syndrome, skeletal muscle, supplementation, white matter

## Abstract

Dietary creatine has been recently put forward as a possible intervention strategy to reduce post‐COVID‐19 fatigue syndrome yet no clinical study so far evaluated its efficacy and safety for this perplexing condition. In this parallel‐group, randomized placebo‐controlled double‐blind trial, we analyzed the effects of 6‐month creatine supplementation (4 g of creatine monohydrate per day) on various patient‐ and clinician‐reported outcomes, and tissue creatine levels in 12 patients with post‐COVID‐19 fatigue syndrome. Creatine intake induced a significant increase in tissue creatine levels in vastus medialis muscle and right parietal white matter compared to the baseline values at both 3‐month and 6‐month follow‐ups (*p* < .05). Two‐way analysis of variance with repeated measures revealed a significant difference (treatment vs. time interaction) between interventions in tissue creatine levels (*p* < .05), with the creatine group was superior to placebo to augment creatine levels at vastus medialis muscle, left frontal white matter, and right parietal white matter. Creatine supplementation induced a significant reduction in general fatigue after 3 months of intake compared to baseline values (*p* = .04), and significantly improved scores for several post‐COVID‐19 fatigue syndrome‐related symptoms (e.g., ageusia, breathing difficulties, body aches, headache, and difficulties concentrating) at 6‐month follow‐up (*p* < .05). Taking creatine for 6 months appears to improve tissue bioenergetics and attenuate clinical features of post‐COVID‐19 fatigue syndrome; additional studies are warranted to confirm our findings in various post‐COVID‐19 cohorts.

## Graphical abstract

Taking creatine for 6 months appears to improve tissue bioenergetics and attenuate clinical features of post‐COVID‐19 fatigue syndrome, possibly due to its energy‐replenishing and neuroprotective activity. Endorsing creatine might be thus of great importance in tackling highly prevalent PFVS after COVID‐19 pandemics.

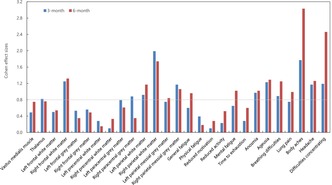

## 1 INTRODUCTION

Post‐viral fatigue syndrome (PVFS) is a perplexing long‐term neurological disorder, formerly known as chronic fatigue syndrome or myalgic encephalomyelitis. PVFS is characterized by an inability to participate in routine activities that were possible before becoming ill, lasting for more than 6 months, and accompanied by fatigue, post‐exertional malaise, and unrefreshing sleep. Although its etiology remains largely unelucidated, PVFS could occur after a viral infection (Morris et al., 2016). Besides other strains, coronaviruses are often linked with PVFS (Chrousos & Kaltsas, 2005; Lam et al., 2009). The current pandemic caused by a member of the coronavirus family severe acute respiratory syndrome coronavirus 2 (SARS‐CoV‐2) could, therefore, lead to many cases of post‐coronavirus disease 2019 fatigue syndrome (Post‐COVID‐19 fatigue syndrome) (Townsend et al., 2020). In line with this, several recent studies demonstrated a relatively high occurrence of PVFS after COVID‐19 (Alkodaymi et al., 2022; Komaroff & Bateman, 2021; Premraj et al., 2022; Simani et al., 2021), with the PVFS‐related symptoms found in up to 45% of COVID‐19 survivors. Developing an effective and convenient intervention strategy to reduce post‐COVID‐19 fatigue syndrome thus becomes of utmost importance to bring down a high disease burden. Among other candidates, dietary creatine has been recently put forward as a possible therapeutic in post‐COVID‐19 recovery having in mind its beneficial effects demonstrated during rehabilitation in various lung conditions (Ostojic, 2020). Since PVFS is often accompanied by various irregularities in creatine metabolism (for a detailed review, see Ostojic, 2021), replenishing creatine via dietary supplementation could be a safe and inexpensive method of nutritional care for post‐COVID‐19 fatigue syndrome. In particular, creatine can help individuals cope with PVFS through several means, including the facilitation of cellular bioenergetics, glutamatergic modulation, neuroprotection, antioxidant activity, and inflammation suppression, domains often compromised in syndromes with chronic fatigue (Ostojic, 2021). A recent trial reported reduced tissue creatine levels in patients with post‐COVID‐19 fatigue syndrome (Ranisavljev et al., 2023), implying a need for replenishing creatine by exogenous administration. Interestingly, creatine and creatine analogues were found effective in various syndromes with long‐term fatigue, including fibromyalgia (Alves et al., 2013) and chronic fatigue syndrome of unknown source (Ostojic et al., 2016), yet no human studies so far have evaluated its effectiveness and safety in the clinical context post COVID‐19. Therefore, the main aim of this randomized controlled trial was to evaluate the effects of medium‐term creatine supplementation on patient‐ and clinician‐reported outcomes, and tissue creatine levels in patients with post‐COVID‐19 fatigue syndrome. We hypothesized that a conventional dosage of creatine might be effective and risk‐free to back up rehabilitation and restore tissue creatine levels of patients with post‐COVID‐19 fatigue syndrome.

## 2 METHODS

### 2.1 Trial design

The study employed a parallel‐group, randomized placebo‐controlled double‐blind design. The allocation ratio to the experimental group (creatine) and control group (placebo) was set at 1:1. No changes to methods were taken after the trial commencement.

### 2.2 Participants

The eligibility criteria for patients to be included in the trial were: age 18–65 years, COVID‐19‐positive test within last 3 months (as documented by valid polymerase chain reaction [PCR] or antigen test performed in COVID‐19 certified lab), moderate‐to‐severe fatigue (e.g., 20‐MFI total test score >43.5), and at least one of additional COVID‐19‐related symptoms, including anosmia, ageusia, breathing difficulties, lung pain, body aches, headaches, and difficulties concentrating. Exclusion criteria were other pulmonary and cardiovascular conditions, and history of dietary supplement use during the past 4 weeks. The eligible participants voluntarily signed informed consent, with the ethical approval obtained by the local IRB at the University of Novi Sad (#46–06‐03/2022A‐2). The study was conducted in compliance with the Declaration of Helsinki (7th revision), and the International Conference of Harmonization Efficacy Guidelines E6. The data were collected at Applied Bioenergetics Lab at the University of Novi Sad, from October 2021 to May 2022.

### 2.3 Interventions

The experimental (creatine) group received 4 g of creatine monohydrate per day, while the control group (placebo) received an equivalent amount of inulin. The dosage of creatine was selected as the standard risk‐free dosage administered across various health domains (Antonio et al., 2021), with the absolute dosage selected for more practical application. The participants were asked to take the intervention once per day during breakfast, with experimental or control powder stirred in 250 mL of lukewarm water and consumed immediately afterward. Both interventions were similar in appearance, texture, and sensory characteristics. Creatine monohydrate was provided by Alzchem GmbH (Creavitalis™, Trostberg, Germany). The duration of the intervention was 6 months, and participants were asked to refrain from using any other dietary supplements and keep regular diet during the trial. A six‐month supplementation period was selected in line with the recommendations for clinical use of creatine in neuromuscular and neurometabolic disorders (Tarnopolsky, 2007).

### 2.4 Outcomes

A list of prespecified primary and secondary outcome measures included five dimensions of fatigue, tissue levels of creatine, patient‐reported outcomes, clinical‐reported exercise tolerance (walking time to exhaustion), and side effects prevalence and severity. All outcome measures were determined at baseline (pre‐administration), after 3 months, and at 6‐month follow‐up (post‐administration). The primary outcome was the change in vastus medialis creatine levels at baseline and 6‐month follow‐up. Fatigue was evaluated with a self‐report pencil‐and‐paper Multidimensional Fatigue Inventory (MFI‐20) test (Smets et al., 1995), with five dimensions of fatigue (general fatigue, physical fatigue, reduced motivation, reduced activity, and mental fatigue) separately calculated and scored. Tissue levels of creatine were measured with proton magnetic resonance spectroscopy (1.5T Avanto scanner, Siemens) using matrix head coil in circularly polarized mode, with metabolite spectra in the specific regions of skeletal muscle and brain (vastus medialis muscle, thalamus, frontal, precentral, paracentral, and parietal white and gray matter) processed as previously described (Ostojic et al., 2016). The patient‐reported outcomes for COVID‐19‐related signs and symptoms (e.g., anosmia, ageusia, breathing difficulties, lung pain, body aches, headache, and difficulties concentrating) were evaluated with the visual analogue scale (VAS) scores. Time to exhaustion was evaluated by an incremental test until exhaustion on a motorized treadmill (speed and gradient of the treadmill are increased every third minute, starting at 1.7 miles per hour at a 10% gradient and rising at stage seven to 6 miles per hour at a 22% gradient) (Will & Walter, 1999). The participants were also asked to report any side effects (e.g., stomach upset, constipation, diarrhea, nausea, vomiting) of either intervention during the study through an open‐ended questionnaire. No changes to trial outcomes appeared after the trial commenced.

### 2.5 Sample size

The minimal sample size (*n* = 12) was calculated prior to the study using power analysis (G*Power 3.1.9.3, Heinrich‐Heine‐Universität Düsseldorf), with the effects size set at 0.50 (medium effect), alpha error probability 0.05, power 0.80, for two groups and three measurements of study outcomes. A medium effect was considered based on previous research assessing the change in vastus medialis creatine levels after creatine intervention (Ostojic et al., 2019). Termination criteria included severe adverse events of the intervention or significant health status changes due to other reasons. A stratified randomization model has been used to achieve balance among groups in terms of subjects' baseline characteristics, with a separate block generated for gender (men and women). After all participants were identified and assigned to the block, simple randomization was performed within each block to assign subjects to one of the interventional groups (creatine and control group). The random allocation concealment was implemented by using sequentially numbered sealed envelopes. The random allocation sequence was generated by a computer program, and a person not related to the study assigned participants to the interventions. Neither participants nor the investigators were aware of the treatment assignment until the end of the study.

### 2.6 Statistical methods

Initially, data were analyzed with the Shapiro–Wilk test for the normality of distribution and Bartlett's test for the homogeneity of the variances. When homogenous variances were verified for normally distributed data, summary measures for interaction effect (time vs. intervention) were compared by two‐way analysis of variance (ANOVA) with repeated measures. When non‐homogenous variances were identified, data were compared using Friedmann's test. Post hoc least significance difference (LSD) test and Wilcoxon test were used to identify differences between individual sample pairs for 2‐way ANOVA and Friedmann's test, respectively. The significance level was set at *p* ≤ .05. Effect sizes after the intervention were assessed by Cohen statistics, with *d* ≥ 0.8 indicating a large effect. The missing data were removed from any analyses. The data were analyzed using the statistical package SPSS version 24.0 for Mac (IBM SPSS Statistics).

## 3 RESULTS

The number of participants who were randomly assigned, received intended treatment, and were analyzed for the primary outcome was 12 in total (six in the experimental group and six in the control group); no losses and exclusions after randomization were reported for either intervention. Baseline demographic characteristics for each group are depicted in Table 1. No differences were found in baseline characteristics between the experimental and control group (*p* > .05).

**TABLE 1:** Baseline demographic characteristics of the study participants.

	Creatine (*n* = 6)	Placebo (*n* = 6)
Age, years, mean ± SD	31.7 ± 9.4	23.3 ± 2.0
Female, *n* (%)	3 (50.0)	3 (50.0)
Weight, kg, mean ± SD	68.5 ± 22.9	72.6 ± 11.8
Height, cm, mean ± SD	172.4 ± 13.5	171.9 ± 9.5
Body mass index, kg/m^2^, mean ± SD	22.5 ± 4.5	24.5 ± 3.1

The changes in primary and secondary outcomes during the trial for each group are shown in Table 2. Creatine intake induced a significant increase in tissue creatine levels in vastus medialis muscle and right parietal white matter compared to the baseline values at both 3‐ and 6‐month follow‐ups (*p* < .05); no changes in tissue creatine values were found in the placebo group throughout the trial. Two‐way ANOVA with repeated measures revealed a significant difference (treatment vs. time interaction) between interventions in tissue creatine levels (*p* < .05), with the creatine group superior to placebo to augment creatine levels at vastus medialis muscle, left frontal white matter, and right parietal white matter. In addition, a non‐significant trend for interaction effect between interventions was reported for several other locations, including right frontal white matter, right paracentral gray matter, left parietal white matter, and left parietal mesial gray matter (*p* ≤ .20).

**TABLE 2:** Tissue creatine levels and patient‐reported outcomes during the trial.

	Creatine (*n* = 6)			Placebo (*n* = 6)			*p* ^a^
	Baseline	3 months	Follow‐up	Baseline	3 months	Follow‐up	
*Total creatine* (*mM*)							
Vastus medialis muscle	28.2 ± 3.0	30.0 ± 4.2^b^	30.6 ± 3.4^b^	20.6 ± 1.9	21.1 ± 3.1	21.9 ± 4.4	<.01
Thalamus	5.7 ± 0.5	6.3 ± 0.9	6.3 ± 1.0	5.9 ± 1.5	6.1 ± 0.8	6.0 ± 1.0	.77
Left frontal white matter	5.9 ± 0.6	6.2 ± 0.6	6.2 ± 0.5	5.8 ± 0.6	5.7 ± 0.6	5.7 ± 0.4	.01
Right frontal white matter	5.6 ± 0.8	6.6 ± 0.8	6.8 ± 1.0	5.7 ± 0.8	5.6 ± 0.7	5.6 ± 0.4	.20
Left frontal gray matter	6.6 ± 0.4	6.9 ± 0.7	6.8 ± 0.7	6.2 ± 0.7	6.2 ± 1.1	6.2 ± 0.8	.94
Right frontal gray matter	6.5 ± 0.7	6.2 ± 0.3	6.2 ± 0.5	6.4 ± 0.7	6.3 ± 0.7	6.3 ± 0.6	.72
Left precentral white matter	6.0 ± 0.6	6.2 ± 0.8	6.1 ± 0.7	5.8 ± 0.4	5.8 ± 0.3	5.9 ± 0.4	.94
Right precentral white matter	5.8 ± 0.5	5.8 ± 0.7	6.0 ± 0.7	5.8 ± 0.3	5.8 ± 0.3	5.9 ± 0.2	.78
Left paracentral gray matter	6.7 ± 0.6	7.3 ± 0.9	7.1 ± 0.7	6.5 ± 0.4	6.3 ± 0.6	6.4 ± 0.7	.31
Right paracentral gray matter	6.8 ± 0.4	7.2 ± 0.5	7.0 ± 0.7	6.7 ± 0.4	6.8 ± 0.4	6.6 ± 0.4	.20
Left parietal white matter	5.3 ± 0.6	6.0 ± 0.9	6.0 ± 0.6	5.2 ± 0.4	5.1 ± 0.4	5.0 ± 0.3	.12
Right parietal white matter	5.3 ± 0.7	6.9 ± 0.9^b^	6.8 ± 1.0^b^	5.1 ± 0.6	5.2 ± 0.8	5.3 ± 0.5	.01
Left parietal mesial gray matter	7.2 ± 0.9	8.0 ± 1.2	8.0 ± 1.0	7.0 ± 0.7	7.0 ± 0.8	7.0 ± 0.9	.06
Right parietal mesial gray matter	7.1 ± 0.3	8.2 ± 1.3	8.1 ± 1.3	7.1 ± 0.4	7.2 ± 0.5	7.1 ± 0.3	.23
*Fatigue* (*score*)							
General fatigue	12.0 ± 0.9	11.3 ± 1.4^b^	12.7 ± 0.5	12.0 ± 0.6	11.8 ± 0.8	12.8 ± 1.0	.74
Physical fatigue	13.5 ± 2.0	12.7 ± 2.1	13.2 ± 1.2	12.5 ± 1.4	12.7 ± 0.5	12.8 ± 1.6	.64
Reduced motivation	13.0 ± 1.4	13.0 ± 1.7	12.5 ± 2.1	12.2 ± 1.2	13.0 ± 2.0	13.7 ± 0.8^b^	.18
Reduced activity	11.5 ± 2.5	12.0 ± 1.9	12.5 ± 1.1	12.7 ± 1.8	12.8 ± 2.9	14.0 ± 1.7	.63
Mental fatigue	12.3 ± 2.0	11.2 ± 1.3	10.5 ± 1.5	11.0 ± 1.8	11.3 ± 1.5	12.2 ± 1.2	.26
*Time to exhaustion* (*sec*)	894 ± 121	922 ± 71	959 ± 94	879 ± 81	883 ± 51	897 ± 91	.75
*Symptoms* (*points*)							
Anosmia	5.0 ± 5.5	1.0 ± 2.0	0.8 ± 2.0	2.9 ± 4.3	1.0 ± 3.2	0.4 ± 1.3	.03
Ageusia	5.0 ± 5.5	0.2 ± 0.4	0.0 ± 0.0^b^	2.0 ± 3.3	0.0 ± 0.0	0.0 ± 0.0	.02
Breathing difficulties	2.3 ± 2.6	0.5 ± 1.2	0.0 ± 0.0^b^	2.9 ± 3.6	1.3 ± 2.5	0.0 ± 0.0	.01
Lung pain	2.3 ± 3.3	0.5 ± 0.8	0.0 ± 0.0	2.0 ± 2.9	0.2 ± 0.4	0.0 ± 0.0	.02
Body aches	4.5 ± 2.1	1.2 ± 1.6^b^	0.0 ± 0.0^b^	4.2 ± 3.6	1.3 ± 2.3^b^	0.7 ± 1.9	<.01
Headache	3.8 ± 3.5	0.8 ± 1.0	0.5 ± 1.2^b^	4.4 ± 3.6	2.2 ± 2.7	0.0 ± 0.0^b^	.01
Difficulties concentrating	4.0 ± 2.3	1.5 ± 1.9^b^	0.0 ± 0.0^b^	2.6 ± 2.8	0.9 ± 1.7	0.2 ± 0.6	<.01

*Note*: Values are mean ± SD.

^a^ Indicates statistical significance for interaction effect (time vs. treatment).

^b^ Indicates a significant difference at *p* = .05 for within‐group comparisons versus baseline values.

Creatine induced a significant reduction in general fatigue after 3 months of intake compared to baseline values (*p* = .04), while the scores for reduced motivation worsened after 6 months in the placebo group (*p =* .02). No significant interaction effect was found between interventions across five MFI‐20 subdomains during the trial (*p* > .05), although a strong trend interaction effect was noted for reduced motivation toward creatine ameliorating this fatigue subdomain and placebo acting in the opposite direction (*p* = .18). Time to exhaustion tended to increase after 6 months of creatine administration (894 ± 121 s at baseline vs. 959 ± 94 s at 6‐month follow up, *p* = .10). Creatine significantly improved VAS scores for several PVFS‐related symptoms compared to baseline values (*p* < .05), except for anosmia and lung pain. The mean scores for body aches and headache were lower at 3‐month and 6‐month post‐administration with placebo (*p* < .05), respectively. In addition, a significant interaction effect was found for all symptoms evaluated (*p* < .05).

**FIGURE 1:**
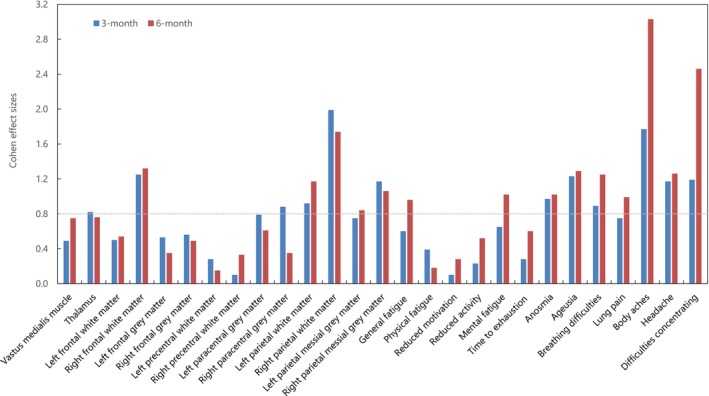
Cohen's effect sizes for primary and secondary outcomes after creatine intervention. The dotted line denotes the effect sizes at 0.80 (large effect).

In addition, the Cohen's effect sizes for primary and secondary outcomes after creatine intake were depicted in Figure 1, with strong effect sizes of creatine (*d* ≥ 0.8) demonstrated for increased brain levels in thalamus (0.82 at 3 months), right frontal white matter (1.25 at 3 months and 1.32 at 6 months), right paracentral gray matter (0.88 at 3 months), left parietal white matter (0.92 at 3 months and 1.17 at 6 months), parietal white matter (1.99 at 3 months and 1.74 at 6 months), left parietal mesial gray matter (0.84 at 6 months), and right parietal mesial gray matter (1.17 at 3 months and 1.06 at 6 months); also for decreasing mental fatigue (1.02 at 6 months), anosmia (0.97 at 3 months and 1.02 at 6 months), ageusia (1.23 at 3 months and 1.29 at 6 months), breathing difficulties (0.89 at 3 months and 1.25 at 6 months), lung pain (0.99 at 6 months), body aches (1.77 at 3 months and 3.03 at 6 months), headache (1.17 at 3 months and 1.26 at 6 months), and difficulties concentrating (1.19 at 3 months and 2.46 at 6 months).

The patients reported no major side effects of either intervention, except for transient mild nausea experienced by one patient after taking creatine. The compliance with the intervention was 90.6% ± 3.5% in the creatine group, and 95.3% ± 5.0% in the placebo group, respectively (*p* = .04).

## 4 DISCUSSION

This is the first human study known to the authors that evaluated the efficacy and safety of **supplemental creatine** for fatigue, tissue bioenergetics, and patient‐reported outcomes in patients with post‐COVID‐19 fatigue syndrome. We found that creatine outcompetes placebo to improve brain and skeletal muscle creatine levels after the medium‐term intervention, and reduces several features of post‐COVID‐10 fatigue syndrome, including lung and body pain, and poor concentration. Creatine induced no major side effects, and might be thus recommended as a safe and effective intervention to tackle post‐COVID‐19 fatigue syndrome.

Several interventional studies evaluated the effects of creatine or creatine analogs in PVFS and similar disorders. Brouwers et al. (2002) evaluated the possible effects of a creatine‐containing polynutrient supplement on several clinical features in patients with chronic fatigue syndrome during a 10‐week interventional interval. The authors found no significant differences between the placebo and the treated group in fatigue severity, functional impairment and other PVFS‐related symptoms. However, the dosage of creatine used in this trial (1.2 g per 100 mL) appears to be insufficient as compared to traditional regimens of creatine supplementation. Another trial investigated the efficacy and safety of creatine supplementation in patients with fibromyalgia, a condition similar to PVFS (Alves et al., 2013). After the 16‐week intervention, the patients receiving creatine (20 g of creatine monohydrate for the first 5 days followed by 5 g per day throughout the trial) exhibited higher muscle phosphocreatine content when compared with the placebo group. This was accompanied by improved mental health, and greater lower‐ and upper body muscle strength, with minor changes found in other fibromyalgia features. A subsequent randomized controlled trial found that a 12‐week supplementation with creatine precursor (guanidinoacetic acid, 2.4 g per day) effectively improves muscle creatine concentrations, functional performance, and specific fatigue subdomains in middle‐aged women with chronic fatigue syndrome (Ostojic et al., 2016).

The present study largely corroborated the results of previous trials demonstrating positive effects of creatine in PVFS and fibromyalgia, while expanding the findings to longer supplementation intervals, and to patients suffering from post‐COVID‐19 fatigue syndrome. We found that creatine significantly increased total creatine levels in several locations across the brain (also a skeletal muscle), with an increase was up to 33% (for the right parietal white matter). Since PVFS is characterized by impaired tissue bioenergetics (Ostojic, 2021), supplemental creatine might be an effective dietary intervention to uphold brain creatine in post‐COVID‐19 fatigue syndrome. This effect might be localized to specific brain regions, with the white matter might be more sensitive to creatine intake in our cohort of patients suffering from post‐COVID‐19 fatigue syndrome. Interestingly, we found here a higher magnitude of brain creatine uptake after the intervention as compared to other neurological conditions (for a review, see Forbes et al., 2022), where brain creatine increases ~5% at post‐administration even when administered in fairly higher dosages as used in our trial. This perhaps suggests a possible disease‐driven susceptibility of the human brain to absorb more creatine in PVFS after COVID‐19. A possible dysregulation of the blood–brain barrier in COVID‐19 (Krasemann et al., 2022) might enable more creatine to be uptaken by the brain and offset the creatine deficit seen in the disease. In addition, an increase in brain creatine was accompanied by improved brain performance after creatine intake, with patients reporting a significant drop of 77.8% in scores for concentration difficulties at the 3‐month follow‐up (Cohen's *d* = 1.19) and no concentration difficulties at the 6‐month follow‐up (Cohen's *d* = 2.46). Also, it seems that creatine in post‐COVID‐19 fatigue syndrome could benefit organs beyond the brain, with the participants in our trial reporting a significant drop in lung and body pain after the intervention, while several other pathognomonic features of post‐COVID‐19 (e.g., anosmia, ageusia, breathing difficulties) also diminished at follow‐up. Possible mechanisms of favorable creatine action in PVFS after COVID‐19 could not only include its role in adenosine triphosphate (ATP) recycling to support energy metabolism in the brain and skeletal muscle but also anti‐inflammatory, antioxidant, and neuromodulatory actions (for a detailed review, see Ostojic, 2021).

Although we used a relatively robust study design, this trial has several limitations. We selected here a sample of young‐to‐middle‐aged adults suffering from moderate post‐COVID‐19 fatigue syndrome; whether creatine is equally effective in other PVFS populations (e.g., elderly, children, patients with less or more severe disease) remains unknown at this moment. Although minor, a difference in age between groups could influence the results. The relatively small size of our sample also restricts identifying possible gender‐related relationships in the data. In addition, we were not able to account for creatine intake from a regular diet that might impact the total net exposure to creatine, as well as for habitual physical activity that could affect tissue uptake of creatine (Harris et al., 1992). Also, we evaluated here the effects of creatine supplementation during a period of 6 months, and a longer administration span might produce different effects. Finally, a more mechanistic approach encompassing a comprehensive set of biomarkers would be needed to address molecular pathways of creatine action in the brain of patients with post‐COVID‐19 fatigue syndrome.

## 5 CONCLUSION

Taking creatine for 6 months appears to improve tissue bioenergetics and attenuate clinical features of post‐COVID‐19 fatigue syndrome, possibly due to its energy‐replenishing and neuroprotective activity. Endorsing creatine might be thus of great importance in tackling highly prevalent PVFS after COVID‐19 pandemics; additional studies are warranted to confirm our findings in various post‐COVID‐19 cohorts.

## AUTHOR CONTRIBUTIONS

**Jelena Slankamenac:** Investigation; Methodology; Validation; Visualization; Writing – review and editing. **Marijana Ranisavljev:** Investigation; Methodology; Validation; Visualization; Writing – review and editing. **Nikola Todorovic:** Investigation; Methodology; Validation; Visualization; Writing – review and editing. **Jelena Ostojic:** Data curation; Formal analysis; Investigation; Methodology; Software; Writing – review and editing. **Valdemar Stajer:** Conceptualization; Data curation; Formal analysis; Investigation; Methodology; Supervision; Validation; Writing – review and editing. **Sergej M. Ostojic:** Conceptualization; Data curation; Formal analysis; Investigation; Methodology; Project administration; Resources; Software; Supervision; Visualization; Writing‐original draft; Writing – review and editing.

[correction added on 5 October 2023, after the first online publication: Missing author contributions was added]

## FUNDING INFORMATION

This work was supported by the Provincial Secretariat for Higher Education and Scientific Research (# 142–451‐2597/2021–01/2); and Alzchem GmbH, Trostberg, Germany.

## CONFLICT OF INTEREST STATEMENT

SMO serves as a member of the Scientific Advisory Board on creatine in health and medicine (AlzChem LLC). SMO co‐owns patent “Supplements Based on Liquid Creatine” at European Patent Office (WO2019150323 A1). SMO has received research support related to creatine during the past 36 months from the Serbian Ministry of Education, Science, and Technological Development; Provincial Secretariat for Higher Education and Scientific Research; Alzchem GmbH; ThermoLife International; and Hueston Hennigan LLP. SMO does not own stocks and shares in any organization. JS, MR, NT, JO, and VS declare no known competing financial interests or personal relationships that could have appeared to influence the authorship of this paper.

## ETHICS STATEMENT

The ethical approval to conduct the current study was granted by the local IRB at the University of Novi Sad (#46–06‐03/2022A‐2).

## CONSENT

The informed consent was obtained from all respondents to participate in the study. The research was conducted ethically in accordance with the World Medical Association Declaration of Helsinki.

## Data Availability

All data analyzed are included in this article. Further enquiries can be directed to the corresponding author.
